# The effects of the Two-Week Rule on NHS colorectal cancer diagnostic services: A systematic literature review

**DOI:** 10.1186/1472-6963-6-43

**Published:** 2006-04-03

**Authors:** Kymberley Thorne, Hayley A Hutchings, Glyn Elwyn

**Affiliations:** 1 School of Medicine, University of Wales, Swansea, UK; 2 Centre for Health Services Research, Cardiff University, Cardiff, UK

## Abstract

**Background:**

The Two-Week Rule (TWR) was introduced to ensure that all patients with a suspected colorectal cancer (CRC) saw a hospital specialist within 14 days of an urgent GP referral. Guidelines were available to GPs to facilitate the appropriate TWR referral of patients exhibiting high-risk CRC symptoms.

**Methods:**

We aimed to evaluate the TWR and its CRC detection rate on NHS CRC diagnostic services by performing a literature search and critically appraising the peer-reviewed studies. Only 12 studies were eligible for inclusion. Data was collected and overall results were given as weighted averages.

**Results:**

The studies identified indicated that only 10.3% of patients referred by the TWR were eventually diagnosed with CRC. When examining the referral origin of all CRC patients diagnosed during the time of the studies, 24% had been referred using the TWR, 24.1% were referred as emergency cases, and 52.4% were referred using alternative routes. No evidence was found to indicate that the TWR had resulted in identifying CRC patients at an earlier, more treatable stage of their disease.

**Conclusion:**

The TWR referral system needs to be improved to increase the number of CRC patients referred using this fast track method as they present to their GP. The TWR and new NICE Guidelines for the referral of patients with suspected cancer should be independently evaluated.

## Background

The Government set targets to be achieved by 2000 for NHS Trusts in England and Wales to see patients with suspected cancer within two weeks of an urgent referral by their GP [1], where "suspected" was defined as either a perceived level of probability or a hunch [2]. This was achieved by the implementation of the Two-Week Rule (TWR) for fast tracking suspected cancer referrals from primary to secondary care [3].

To facilitate the appropriate use of the TWR, Guidelines were published by the Department of Health (DoH) detailing the high-risk criteria exhibited by patients with suspected cancer [4]. In the case of colorectal cancer (CRC), the Guidelines' list of high-risk symptoms aimed to "identify up to 90 per cent of patients with bowel cancer" to be urgently referred using the TWR. The Guidelines recommended that a patient needed to exhibit a combination of the high-risk symptoms to be appropriately referred using the TWR. Patients referred using the TWR that did not exhibit at least two of the criteria would be considered inappropriate referrals.

Since 2000, English NHS Trusts have reported their compliance with the TWR to the DoH on a quarterly basis. The report for the first Quarter of 2005/06 showed that 99.5% of all patients referred with a suspected lower gastrointestinal cancer via the TWR were seen within two weeks [5].

A recent publication by Chohan *et al *highlighted the success of the TWR in speeding up patients' access to clinics at their hospital but reported a low CRC detection rate of 27%. The CRC patients diagnosed via this route also tended to have more advanced stage of the disease [6].

In light of this, we performed a literature search to evaluate all peer-reviewed evidence reporting on the TWR and its CRC detection rate within NHS CRC diagnostic services to determine whether the TWR was effective in identifying suspected CRC patients, and whether they were diagnosed at an earlier stage of their disease.

## Methods

A literature search was performed using Medline, the Cochrane Library, the National Library for Health (NLH), the Health Management Information Consortium (HMIC) and the Centre for Reviews and Dissemination (CRD), employing a text search for peer-reviewed research publications. The search terms used were "colorectal" or "CRC" in combination with "urgent referral*", "two week*", "2-week*" and "fourteen day*".

Studies performed outside England and Wales were excluded, as were studies performed prior to the implementation of the TWR in July 2000 or in non-NHS organisations. Only peer-reviewed studies commenting on the effectiveness of the TWR in NHS CRC diagnostic services were selected based on the abstract by one author (KT).

Data describing the TWR CRC detection rate and the referral routes of CRC patients identified during the study were extracted, as well as any reports on the stage of the disease in CRC patients. Data extracted were actual values. All studies with comparable datasets were combined to give overall results, given as weighted averages.

## Results

During the initial search for literature, a total of 123 articles were retrieved. Of these, 103 were immediately excluded because they did not refer to TWR in any way. Of the remaining 20 articles, one did not relate directly to TWR efficiency, four studies were done prior to the official implementation on TWR in July 2000 and three were letters. This left 12 articles suitable for inclusion. Of these, only eight had comparable data reporting on the effect of the TWR in NHS CRC diagnostic services. These studies consisted of two retrospective studies and six prospective studies, four of which were based on audits (see Table 1). These studies were performed between 2000 and 2003.

The studies identified showed that of the 2440 patients referred by their GP using the TWR, only 10.3 per cent were subsequently diagnosed with CRC (see Table 2).

When determining the referral route of all CRC patients diagnosed during the time of the studies identified, we found that only 24 per cent of CRC patients were referred by GPs using the TWR, [6-13] with a further 24.1 per cent being referred as emergency cases [6-8,11-13]. The remaining 52.4 per cent had been referred by other routes (see Table 2) [6-8,11-13]. For two studies, [9,10] the number of emergency cases could not be extracted from the non-TWR referrals and were not included in the summary values above.

Although most patients were seen by a hospital specialist within the two-week target, no studies reported any significant difference in the stage of the disease of CRC patients referred and diagnosed using the TWR compared with other referral routes [6,8,11,14].

Of the four articles retrieved in the literature search but not included in Table 1, one was a systematic review of cancer waiting time audits [15] commissioned by the DoH to inform the new NICE Guidelines [16] and published by the CRD. This review identified a total of 39 hospital audits relating to lower GI cancer. From these, the review reported that the CRC detection rate for the TWR ranged from 2 – 22 per cent and that between 0 – 47 per cent of CRC patients were referred using the TWR.

The remaining three studies retrieved by the literature search included a qualitative assessment of the DoH Guidelines, [14] one focussed on the impact of a fast track barium enema service, [17] and one reported on variations in the evaluation of CRC risk [18].

## Discussion

Although the DoH Guidelines aimed to identify 90 per cent of CRC patients as they presented to their GP, the literature identified by this paper indicated that only 10.3 per cent were eventually diagnosed with CRC. This figure is based on the assumption that all TWR referrals were appropriate. Less than a quarter of all CRC patients had been referred using the TWR, the same proportion had been referred as emergency cases and just over half of all CRC patients diagnosed during the time of the studies had been referred using alternative routes. There was no evidence to indicate any significant improvement in detecting CRC at an earlier stage in CRC patients referred using the TWR compared to those referred by alternative routes.

This paper reviewed all relevant peer-reviewed evidence from studies performed after 2000 reporting on the impact of the TWR. There was a limited amount of peer-reviewed literature in this field, with only 12 publications meeting our inclusion criteria, and only eight of these reporting comparable datasets. A single researcher assessed the literature, but the tight focus on the inclusion and exclusion criteria should have reduced any bias in the selection of eligible studies. As far as we are aware, no other evaluation of the literature in this field has been performed to date.

Our assessment of the guidelines was based on calculating the percentage of TWR-referred patients eventually diagnosed with CRC from all the studies and determining a weighted average. We also compared the proportion of CRC patients referred by the TWR compared with alternative routes into secondary care using weighted averages calculated from the original papers.

The low number of CRC patients identified following a TWR referral suggests that the Guidelines were not as effective in identifying CRC patients as they presented to their GP as was hoped. This may be explained by GPs referring patients who did not conform to the Guidelines, possibly due to the incorrect interpretation of the Guidelines or to intentionally speed up diagnoses in low-risk patients where the routine waiting list was too long.

The studies identified reported that the proportion of CRC patients diagnosed by alternative routes (excluding emergencies) was more than double those referred using the TWR. These patients may not have exhibited any of the high-risk symptoms specified by the Guidelines, or they may have been internally referred following radiological or pathological investigations from within secondary care, where the TWR cannot be applied. Alternatively, it may be the result of the referral practices of GPs [19]. These high numbers of non-TWR referrals are cause for concern and the TWR referral methodology may need to be changed to capture these CRC patients.

Almost a quarter of all CRC patients were diagnosed following an emergency referral, although we were unable to determine whether the source of their referral was primary or secondary care. The majority of CRC patients diagnosed following this route could not have been diverted onto the TWR route if they presented with "emergency" symptoms in need of immediate treatment, especially if they presented to A&E instead of their GP.

The TWR did not result in CRC patients being diagnosed at an earlier, more treatable stage of their disease. This could be because patients correctly referred using the TWR Guidelines need to exhibit high-risk symptoms indicative of later stage CRC, whilst early stage CRC patients may not have any alarm symptoms and may have been referred using alternative routes.

Although CRC lacks any highly specific symptoms [12], Selvachandran et al have successfully used a patient questionnaire to prioritise CRC referrals [20]. The studies identified in this review have shown that compiling highly specific Guidelines for symptomatic CRC patients using TWR referrals without compromising on their ability to detect early CRC can be problematic [8,10,13,14]. A literature search performed by Hamilton and Sharp concluded that the DoH Guidelines were based on a reasonable evidence base [21] but the studies identified for this paper indicate otherwise, although it is worth noting that TWR referrals were assumed to be appropriate in accordance with the Guidelines. Research into the positive predictive value of CRC symptoms in the UK population is ongoing [22] to improve the sensitivity and specificity of the Guidelines, although the revised TWR referral Guidelines by NICE [16], published in 2005, are almost identical to the original DoH Guidelines and so, are not likely to increase the proportion of CRC patients diagnosed using the TWR.

The TWR was rapidly implemented throughout NHS cancer diagnostic services with no pilot studies done to indicate potential problems. Both primary and secondary care services had to cope with the new referral protocols and their subsequent impact on services. We would question whether the TWR has had any positive effect on the identification of CRC patients at an earlier stage of their disease. To address this, we recommend that the TWR and the revised NICE Guidelines should be officially evaluated by an independent group to determine whether there is any subsequent increase in the identification of CRC patients, accompanied by an improvement in the cancer stage at diagnosis to make the TWR worthwhile. A small scale evaluation has already been successfully established [23] and could be used as a basis for a nationwide study. We also recommend that the new NICE Guidelines be made compulsory for all GPs and that TWR referral documentation is standardised so that comparisons can be made during any evaluations.

## Conclusion

The evidence presented in this paper indicates that the detection rate for TWR-referred CRC was low and approximately half of all CRC patients are referred by alternative routes prior to their diagnosis. Although most patients were seen by a hospital specialist within two weeks of referral, the TWR did not result in the identification of CRC patients at an earlier stage of their disease. We conclude that the TWR referral system is in need of improvement and independent evaluation.

## Competing interests

The author(s) declare that they have no competing interests.

## Authors' contributions

KT conceived of the study, performed the literature review and drafted the manuscript. HAH and GE contributed to the design of the study and the literature review and were involved in drafting the manuscript. All authors read and approved the final manuscript.

## Pre-publication history

The pre-publication history for this paper can be accessed here:



## Figures and Tables

**Table 1:** Description of the studies used in this paper.

**First author & Reference**	**Location of study**	**Study Type**	**Time period studied**
Chohan [6]	Addenbrookes Hospital, Cambridge	Retrospective observational study	Jul 2000 to Dec 2001
Maruthachalam [7]	Newcastle upon Tyne NHS Trust	Prospective evaluative study	Jan to Dec 2003
Eccersley [8]	Luton & Dunstable NHS Trust	Prospective audit	Jan 2000 to March 2001
Walsh [9]	Blackpool Victoria Hospital, Blackpool	Prospective audit	Aug to Oct 2000
Debnath [10]	South Durham Healthcare Trust	Prospective audit	Aug 2000 to Jul 2001
Flashman [11]	Queen Alexandra Hospital, Portsmouth	Prospective audit	Jul 2000 to Jun 2001
Barwick [12]	St James' University Hospital, Leeds	Retrospective observational study	Jan to Aug 2001
Tricket [13]	Ashford & St Peter's Hospital NHS Trust	Prospective observational study ^†^	Nov 2000 to Oct 2001

Key: ^† ^Study looked at CRC patients only

**Table 2:** Data from eight peer-reviewed studies describing the effect of the TWR on their colorectal services.

**First author** ** & Reference**	**N° TWR** ** referrals received**	**(i) % of CRCs identified** ** from TWR referrals (n) **	**Total number of CRC** ** cases diagnosed †**	**Total % of CRC cases** ** referred as...**		
				**TWR**	**Emergency**	**Other**
Chohan [6]	462	13.8% (64)	195	32.8%	20%	47.2%
Maruthachalam [7]	639	8% (51)	234	21.8%	10.6%	67.6%
Eccersley [8]	180	14.4% (26)	145	18%	17%	66%
Walsh [9]	78	10.3% (8)	23	47.8%	52.2%	
Debnath [10]	237	8.9% (21)	96	21.9%	78.1%	
Flashman [11]	695	9.4% (65)	249	26.1%	35.3%	38.6%
Barwick [12]	149	10% (14)	84	16.7%	41.7%	41.7%
Tricket [13]	NA	NA	147	20%	29%	51%
**Weighted averages of all values combined**		**10.3%**		**24%**	**24.1%**	**52.4%**

Key: NA = Data not available in article; † = Total number of CRC cases diagnosed during the time of the study in the same locality.
